# Cancer‐associated fibroblasts in nonsmall cell lung cancer: From molecular mechanisms to clinical implications

**DOI:** 10.1002/ijc.34127

**Published:** 2022-06-08

**Authors:** Kit Yee Wong, Alvin Ho‐Kwan Cheung, Bonan Chen, Wai Nok Chan, Jun Yu, Kwok Wai Lo, Wei Kang, Ka Fai To

**Affiliations:** ^1^ Department of Anatomical and Cellular Pathology, State Key Laboratory of Translational Oncology Prince of Wales Hospital, The Chinese University of Hong Kong Hong Kong SAR China; ^2^ Institute of Digestive Disease, State Key Laboratory of Digestive Disease, The Chinese University of Hong Kong Hong Kong SAR China; ^3^ Li Ka Shing Institute of Health Science, Sir Y.K. Pao Cancer Center, The Chinese University of Hong Kong Hong Kong SAR China; ^4^ Department of Medicine and Therapeutics The Chinese University of Hong Kong Hong Kong SAR China

**Keywords:** cancer‐associated fibroblast, heterogeneity, nonsmall‐cell lung cancer, tumor microenvironment

## Abstract

Lung cancer is the common and leading cause of cancer death worldwide. The tumor microenvironment has been recognized to be instrumental in tumorigenesis. To have a deep understanding of the molecular mechanism of nonsmall cell lung carcinoma (NSCLC), cancer‐associated fibroblasts (CAFs) have gained increasing research interests. CAFs belong to the crucial and dominant cell population in the tumor microenvironment to support the cancer cells. The interplay and partnership between cancer cells and CAFs contribute to each stage of tumorigenesis. CAFs exhibit prominent heterogeneity and secrete different kinds of cytokines and chemokines, growth factors and extracellular matrix proteins involved in cancer cell proliferation, invasion, metastasis and chemoresistance. Many studies focused on the protumorigenic functions of CAFs, yet many challenges about the heterogeneity of CAFS remain unresolved. This review comprehensively summarized the tumor‐promoting role and molecular mechanisms of CAFs in NSCLC, including their origin, phenotypic changes and heterogeneity and their functional roles in carcinogenesis. Meanwhile, we also highlighted the updated molecular classifications based on the molecular features and functional roles of CAFs. With the development of cutting‐edge platforms and further investigations of CAFs, novel therapeutic strategies for accurately targeting CAFs in NSCLC may be developed based on the increased understanding of the relevant molecular mechanisms.

## Graphical abstract

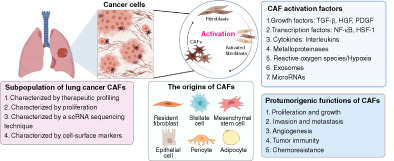

**Abbreviations**
ATF6: activating transcription factor 6
C3a: CAF‐secreted complement 3a
CAFs: cancer‐associated fibroblasts
CCL: C‐C chemokine ligand
CF: core fucosylation
CLCF1: ardiotrophin‐like cytokine factor
CRBPD: CCAAT/enhancer‐binding protein delta
CSF1: colony‐stimulating factor 1
CXCL: CXC motif chemokine ligand
ECM: extracellular matrix
ECs: endothelial cells
ECs: epithelial cells
EGFR‐TKIs: EGFR tyrosine kinase inhibitors
EndET: endothelial‐to‐mesenchymal transition
FAP‐1: Fas‐associated phosphatase 1
FGF: fibroblast growth factors
FSP1: fibroblast specific protein 1
Fut8: fucosyltransferase 8
GFs: growth factors
GGT5: gamma‐glutamyl transferase 5
HA: hyaluronic acid
HD‐CAF: high desmoplastic CAFs
HGF: hepatocyte growth factor
Hh: hedgehog
HIF‐1α: hypoxia‐inducible factor‐1α
HMGB1: high mobility group box 1
HOTAIR: HOX transcript antisense RNA
HSCs: hepatic stellate cells
IGF‐1: insulin‐like growth factor
IL: interleukin
LD‐CAF: low desmoplastic CAFs
LIF: leukemia inhibitory factor
lncRNA: long noncoding RNA
LOXL1: lysyl oxidase‐like 1
LUAD: lung adenocarcinoma cells
MDSCs: myeloid‐derived suppressor cells
miRNAs/miRNA: microRNAs
MMP‐2: matrix metalloproteinase‐2
MRTF: myocardin‐related transcription factors
MSCs: mesenchymal stem cells
MSCs: mesenchymal stromal cells
MT: epithelial to mesenchymal transition
NFs: normal fibroblasts
NK: natural killer
Nrf2: nuclear factor erythroid 2‐related factor 2
NSCLC: nonsmall cell lung carcinoma
PDGF: platelet‐derived growth factor
PDPN: podoplanin
PSCs: pancreatic stellate cells
ROS: reactive oxygen species
SDF‐1: stromal cell‐derived factor‐1
SHh: sonic hedgehog
SMO: 7‐transmembrane protein smoothened
SRGN: sulfate proteoglycan serglycin
STC‐1: stanniocalcin‐1
TAMs: tumor‐associated macrophages
TANs: tumor‐associated neutrophils
TGF‐β: transforming growth factor‐beta
TGF‐βR: transforming growth factor‐β receptor
TIAM2: T‐cell lymphoma invasion and metastasis 2
TIME: tumor immune microenvironment
TME: tumor microenvironment
TNF‐α: tumor necrosis factor‐α
VCAM1: vascular cell adhesion molecule‐1
VEGF: vascular endothelial growth factor
αSMA: alpha‐smooth muscle actin

## 1 INTRODUCTION

Lung cancer is one of the most common malignancies worldwide, particularly in men.^1^ The American Cancer Society reported approximately 2.2 million new cases of lung cancer and approximately 1.8 million new deaths in 2020.^1^ Lung cancer is histologically classified as small‐cell lung carcinoma (SCLC) and nonsmall‐cell lung carcinoma (NSCLC). NSCLC represents approximately 80% of all lung cancer^2^ and is mainly divided into squamous cell carcinoma, adenocarcinoma and large cell carcinoma. These subtypes have unique histological and biological features.^3^, ^4^ Enhancing insight into the genome alterations revealed various oncogenic driver mutations in NSCLC.^5^, ^6^

To understand the biological perspectives of lung cancer, researchers have mainly focused on malignant cells, such as various signaling pathways.^7^ However, these just represent one of the hallmarks of cancer. Cancers are not simply composed of cells with deranged signaling pathways but include a complex tumor microenvironment (TME).^8^, ^9^, ^10^ Like the theory of “Seed and Soil”^11^ which was proposed by Dr Stephen Paget, cancers cells seed in congenial soil, the TME, where they grow and expand. The TME is an ecosystem composed of multicellular and noncellular components.^12^, ^13^ Four major components of the TME are: (1) the tumor immune microenvironment (TIME) consists of immune cells such as natural killer (NK) and T cells; (2) vascular components include lymphatic endothelial cells (LECs) and pericytes; (3) the extracellular matrix (ECM) is comprised of diverse collagen, glycoproteins and proteoglycans; (4) stromal components consist of mesenchymal STEM cells (MSCs) and cancer‐associated fibroblasts (CAFs).^14^ These cells in the TME interact with the malignant cells closely, which promote the whole tumorigenesis process, from tumor initiation to progression.

CAFs are one of the well‐known and critical components in the tumor stroma. CAFs are worthy of mention since they are conducive to all aspects of tumorigenesis in different stages and many cancer types, including tumor proliferation, tumor invasion and metastasis and interfacing with the immune system.^14^, ^15^ Given the multifaceted functions of CAFs, many studies attempted to “switch off” the function of CAFs to target tumors more effectively. Controversially, some investigations have demonstrated that some CAFs have an antitumorigenic role.^16^, ^17^ More importantly, how can CAFs transit from a tumor defender into a tumor supporter? For example, tumor‐associated exosomes have been identified recently as an essential cellular interchange mechanism between tumor cells and CAFs.^18^, ^19^ Isolated exosomes from tumor cells and CAFs are implicated in multiple steps of CAFs evolution, such as normal fibroblasts (NFs) differentiation into CAFs, CAF‐like state maintenance and promotion of CAFs' oncogenic properties.^20^, ^21^, ^22^, ^23^ Extracellular vesicles produced by tumor cells can activate normal fibroblasts to a CAF‐like state, which in turn produces a secretome to modulate the tumor microenvironment.^24^, ^25^ In this review, we summarized recent studies on the roles of CAFs and, particularly in NSCLC, where scar formation and fibrosis are common phenomena.

## 2 THE DEFINITION AND BIOLOGICAL PROPERTIES OF CAFs

### 2.1 Fibroblasts

Fibroblasts were first identified in the 1850s as connective tissue cells responsible for synthesizing collagen.^26^ Fibroblasts in normal tissue are generally considered quiescent, that is, in a resting state. Fibroblasts can be challenging to define because of a lack of unique markers expressed exclusively and by all fibroblasts.^27^ Some markers such as vimentin, platelet‐derived growth factor receptor‐α (PDGFR‐α) and fibroblast specific protein 1 (FSP1) can be used as markers for quiescent fibroblasts.^28^, ^29^, ^30^, ^31^ However, these markers are not only expressed in fibroblasts. Thus, the tissue location and morphology are always required for their identification.

Quiescent fibroblasts are the major component of ECM under physiological conditions. They are activated by tissue repair and regeneration in response to tissue damage. As observed in wound healing,^32^ fibroblasts accumulate at the damaged site and transform into myofibroblasts, and subsequently promote angiogenesis and deposition of ECM. Myofibroblasts produce many kinds of cytokines and chemokines.^15^, ^33^ They are also a significant source of ECM‐degrading proteases, maintaining ECM homeostasis by regulation of ECM turnover,^34^ and promoting angiogenesis with increased production of vascular endothelial growth factor A (VEGFA).^35^ Myofibroblasts secrete transforming growth factor‐beta (TGF‐β) and express α‐smooth muscle actin (α‐SMA) at closing wounds,^36^ and are critical for maintaining the homeostasis of adjacent epithelial cells by growth factors (GFs) secretions and by direct mesenchymal‐epithelial cell interactions.^37^ When the wound is healed, myofibroblasts are restored to their quiescent status or are removed by apoptosis.^38^ Such reversibility is a hallmark feature of fibroblasts associated with tissue repair.

### 2.2 Activation of fibroblasts into CAFs

Tumors may be considered as “wounds that do not heal.”^26^ In a normal situation, fibroblasts have an antitumorigenic activity that suppresses tumor growth. For example, fibroblasts in lymph nodes transport potential antigens and contribute to leukocytes' migration, resulting in effective immune responses.^17^ However, cancer is an advancing and unabated injurious stimulus which initiates fibroblast activation. Fibroblasts are then transformed into irreversible cancer‐associated fibroblasts (CAFs), which behave like myofibroblasts in some aspects.^39^ They are not removed by apoptosis. This process is called cancer fibrosis.

**FIGURE 1:**
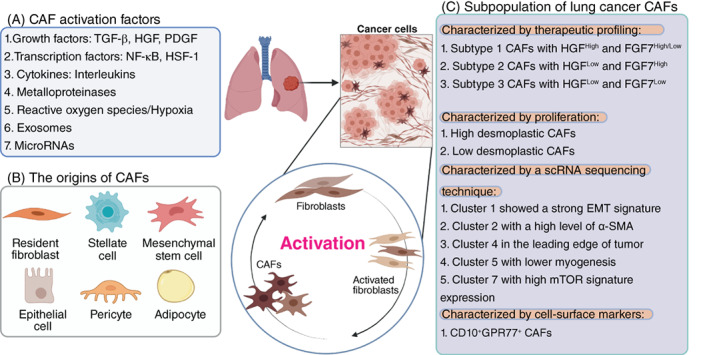
The origin, activating factors and subpopulations of cancer‐associated fibroblasts (CAFs) in nonsmall‐cell lung cancer (NSCLC) are diverse and heterogeneous. (A) Multiple activating factors promote the transition from normal fibroblasts to CAFs in the tumor microenvironment. (B) The diversity of CAF sources. CAFs are not only derived from resident fibroblasts, but also from other cell types, including stellate cell, mesenchymal stem cell, epithelial cell, pericyte and even adipocyte. (C) Various classification systems for lung cancer CAFs to define subpopulations based on molecular features and functional roles (created with BioRender.com) [Color figure can be viewed at wileyonlinelibrary.com]

To acquire tumor‐promoting phenotypes, the quiescent fibroblasts are activated via diverse mechanisms (Figure 1A). First, epithelial cancer cells secrete growth factors into the surrounding microenvironment, stimulating the recruitment and activation of fibroblasts. Among these factors, transforming growth factor‐beta (TGF‐β), platelet‐derived growth factor (PDGF) and fibroblast growth factor (FGF) are critical regulators. In lung cancer, TGF‐β facilitates invasion of cancer cells through tumor‐stromal interactions.^40^, ^41^ TGF‐β orchestrates tumor stroma development and promotes angiogenesis, immune evasion and remodeling of the ECM.^42^, ^43^ In microarray gene expression analysis, the gene signatures related to TGF‐β signaling are enriched in CAFs isolated from NSCLC tissues compared to the normal tissue.^44^ PDGF is one of the profibrotic growth factors secreted by cancer and stromal cells, inducing CAFs activation.^45^, ^46^ Cancer cells secrete PDGF to act on the stromal cells, especially endothelial cells and fibroblasts in vivo.^47^ In contrast to TGF‐β, the primary functions of PDGF are enhancing fibroblasts' growth and proliferation through MAPK downstream signaling pathways^48^, ^49^, ^50^ without causing their differentiation into myofibroblasts.^51^ PDGF is also a crucial factor in neo‐angiogenesis and establishing protumorigenic stroma.^45^, ^52^ FGF, an angiogenic endothelial cell mitogen, is a pleiotropic molecule that functions on epithelial and mesenchymal cells in an intracrine, autocrine and paracrine manner.^53^, ^54^ Most studies focused on FGF‐2, which describes how it changes the phenotype of fibroblasts, leading to cell activation.^53^

Besides growth factors, lung cancer cells produce different inflammatory modulators such as the interleukin family (IL‐6, IL‐8, IL‐17, IL‐22), tumor necrosis factor‐α (TNF‐α) and VEGF to promote their progression, invasion and angiogenesis.^55^ Many studies found that these inflammatory cytokines are related to fibroblast activation in lung cancer. Leukemia inhibitory factor (LIF), an IL‐6 class proinflammatory cytokine, is an example. It mediates ECM remodeling and TGF‐β‐dependent actomyosin‐contractility via crosstalk between the JAK1/STAT3 and RhoA/ROCK/MLC2 signaling pathways, which results in carcinoma cell invasion in vitro and in vivo.^56^ Actomyosin contractility generates mechanical force to remodel the ECM for cell migration, which is caused by CAFs.^57^ The roles of STAT3 and SMAD are also implicated in lung fibrosis.^58^

In NSCLC, the oxygen level is deficient (0.7‐46 mm Hg),^59^ thus hypoxia is a characteristic of the lung cancer microenvironment. This remodels the composition of TME, and induces the expression of HIF‐1α in fibroblasts.^60^, ^61^, ^62^, ^63^ The expression of HIF‐1α in fibroblasts also induces the conversion of normal fibroblasts into CAFs, and CAFs activation can be inhibited effectively by HIF‐1α‐specific inhibitors or HIF‐1α knockout.^64^ Moreover, p62, an autophagy regulator, is highly expressed in NSCLC under hypoxia.^65^ This induces autophagy, the nuclear factor erythroid 2‐related factor 2 (Nrf2)‐related antioxidant signaling, and the activating transcription factor 6 (ATF6)‐related ER‐stress response, causing CAFs activation. Autophagy inhibitors such as 3MA and HCQ can block CAF activation and tumor progression, supporting the critical role of p62‐dependent autophagy in CAFs activation.^66^ While Nrf2 is persistently elevated, fibroblasts are induced into a state of increased activity and acquire the CAF phenotype, leading to tumor growth.^67^

MicroRNAs (miRNAs) are small endogenous noncoding RNAs that mainly downregulate target gene expression^68^, ^69^, ^70^ and are potential biomarkers in cancer patients.^71^, ^72^, ^73^ miRNAs also contribute to the activation of CAFs during cancer progression. Previous studies demonstrated that some miRNAs are highly expressed in lung adenocarcinoma and promote CAFs activation, such as miR‐196a,^74^ miR‐210^75^ and miR‐21.^76^ Moreover, some miRNAs are highly expressed in NF in lung fibrosis and alter the phenotype of primary fibroblasts, such as miRNA‐155^77^ and Let‐7d miRNAs.^78^ These findings suggest that miRNAs can have a regulatory role in transforming NFs to CAFs in lung adenocarcinoma.

Besides posttranscriptional control, epigenetic mechanisms are also implicated in CAF activation.^68^, ^69^, ^79^, ^80^ A study showed that the proinflammatory cytokine leukemia inhibitory factor (LIF) enhanced the invasive potential of CAFs, by mediating the acetylation of STAT3 by histone acetyltransferase p300, and subsequently the activation of JAK1 by promoter methylation of SHP1.^79^ Recently, an interesting study highlighted that CAFs transactivated the lncRNA HOX transcript antisense RNA (HOTAIR) expression in breast cancer cell via the secretion of TGF‐β1, and also histone H3K27 trimethylation to activate the CDK5 pathway, contributing to cancer metastasis and EMT.^80^ More epigenetic control nodes in CAFs activation are expected to be unraveled.

Many studies reported exosomes as the messengers participating in the crosstalk between cancer cells and fibroblasts in promoting CAFs activation.^24^, ^81^ Exosomes are lipid‐bilayer extracellular nanovesicles carrying microRNAs,^25^, ^75^, ^82^ lncRNAs,^83^ proteins,^21^, ^84^ metabolites and other substances.^85^, ^86^ Zhang et al determined the protein secretome of fibroblasts treated with or without cancer cell‐derived exosomes. They found that cancer cells and fibroblasts have a bidirectional interaction; Cancer cell‐derived exosomes activate fibroblasts into CAFs while CAFs secreted proteins enhances proliferation and migration of NSCLC cells.^87^

## 3 THE HETEROGENEITY OF CAFs

### 3.1 The origin of CAFs

Emerging evidence suggests that CAFs are a highly heterogeneous population of cells.^16^ Such heterogeneity might be due to the numerous potential cellular sources and precursors of CAFs (Figure 1B).^88^, ^89^ NFs can be activated by the TME stimuli, such as local hypoxia, oxidative stress and GFs released from the neighboring tumor cells and infiltrating immune cells. This theory suggests that CAFs can be derived from resident fibroblasts activated by adjacent tumor cells and are the primary source of CAFs.^90^ TGF‐β, secreted by the stromal and cancer cells, promotes the migration of resident fibroblasts and their transformation into CAFs. In some organs, such as the pancreas and liver, the resident fibroblasts are known as quiescent pancreatic stellate cells (PSCs) and hepatic stellate cells (HSCs), respectively.^91^, ^92^ They can acquire a myofibroblast‐like phenotype such as α‐smooth muscle actin (α‐SMA) expression upon TGF‐β and PDGF activation. Furthermore, some CAFs can transdifferentiate from mesenchymal stem cells (MSCs).^93^, ^94^ CXCL‐12 and TGF‐β, which are secreted by tumor cells, stimulate the recruitment of MSCs and their activation into CAFs.^16^, ^95^, ^96^ Bone marrow derived (BM)‐MSCs show upregulation in Calponin 1, a‐SMA and collagens by myocardin‐related transcription factors (MRTF) to induce differentiation into CAFs.^88^, ^97^, ^98^

Studies also suggested that pericytes,^99^, ^100^ smooth muscle cells surrounding small border vessels can transdifferentiate into CAFs. Pericytes have been considered an essential source of myofibroblasts.^101^, ^102^ The process starts with pericyte detachment from endothelial cells, followed by migration into the lung interstitium and then activation to become myofibroblast via TGF‐β/Smad2/3 and PDGFβ/Erk signaling pathways.^103^, ^104^, ^105^ Here, the transforming growth factor‐β receptor (TGF‐βR) and platelet‐derived growth factor‐β receptor (PDGF‐βR) are modified by core fucosylation (CF). α1,6‐fucosyltransferase (FUT8) is the only known enzyme that catalyzes CF.^106^, ^107^ In FUT8 knockdown cells, CF is out of function, and this inhibits TGF‐β/Smad2/3 and PDGFβ/Erk signaling pathways.^108^ Some studies suggested that Sonic Hedgehog (SHh) is also involved in CAFs activation.^109^, ^110^ SHh contributes to branching morphogenesis lung specification in the developing lung.^111^ In normal conditions, Hedgehog (Hh) activity is low. In the context of bleomycin injury, lung damage induces Hh pathway activity, and SHh overexpression increases fibrotic collagen deposition.^112^ In idiopathic pulmonary fibrosis, Hh activity can promote multiple profibrotic processes, including enhanced sensitivity to TGFβ and PDGF, leading to increased migration, contractility and survival in human lung fibroblasts.^113^, ^114^

CAFs can also derive from epithelial cells (ECs).^115^ ECs differentiate into functional CAFs, which express FSP‐1 and αFAP via TGF‐β‐mediated epithelial to mesenchymal transition (EMT).^116^, ^117^ Endothelial cells contribute to the pool of CAFs through endothelial‐to‐mesenchymal transition (EndMT) in cancer, mainly via TGF‐β and SMAD signaling.^118^, ^119^ Several groups have also reported adipocyte conversion into CAFs. Mature adipocytes can activate the Wnt/β‐catenin pathway, leading to adipocyte “dedifferentiation” to acquire a fibroblast‐like morphology.^99^, ^120^, ^121^, ^122^ It is suggested that using lineage tracing method with single cell spatial analysis can find out the main role and function of each cell type in tumor development, thus accounting for CAFs heterogeneity.^123^

### 3.2 Subpopulation of CAFs in NSCLC and other cancers

Determination of subtypes of CAFs has met significant obstacles due to the heterogeneity of their origin, phenotype and function among different individuals in different tumor types. Based on different classification methods, there are different names for different subtypes of CAFs, as shown in Table 1 and Figure 1C. Different classifications have been proposed in relation to the different analysis approaches, for example the immunophenotype, RNA expression profile and histologic findings. In breast cancer, based on spatial distribution, CAFs can be classified as vascular CAFs, matrix CAFs, cycling CAFs and developmental CAFs. These subtypes of CAFs have discrete gene expression profiles. The gene sets detected for vascular CAFs were related to vascular development and angiogenesis, while matrix‐related genes dominated in matrix CAFs. Cycling CAFs are the proliferating section of vascular CAFs and are enriched for gene sets of the cell cycle. Lastly, differentiation‐related genes were hallmarks of developmental CAFs.^124^

**TABLE 1:** The proposed classification of CAFs in breast, pancreatic and lung cancer

Classification methods	Cancer type	Origin or function	Proposed CAF subtype	References
Spatial distributions	Breast cancer	Originate from peripheral blood vessels	Vascular CAFs	124
		Originate from resident fibroblasts in local tissues	Matrix CAFs	
		Proliferating section of vascular CAFs	Cycling CAFs	
		Similar in phenotype to tumor epithelial cells	Developmental CAFs	
Biomarkers	Breast cancer	Highly express basement membrane protein,	CD146^+^ CAFs	125
		Highly express products that promote tumor invasiveness	CD146^−^ CAFs	
Phenotypes	Pancreatic cancer	Myofibroblastic phenotypes	myCAFs	126
		Inflammatory phenotypes	iCAFs	
Functions	Pancreatic cancer	Epithelial‐to‐mesenchymal transition (EMT)	EMT‐CAFs	127
		Proliferation	PRO‐CAFs	
Histological features	Lung cancer	High desmoplastic CAFs	HD‐CAFs	128
		Low desmoplastic CAFs	LD‐CAFs	
Single‐cell RNA sequencing technique	Lung cancer	A strong signature of EMT and clustering with tumor cells	Cluster 1	129
		A high level of α‐SMA and cocluster with pericytes	Cluster 2	
		Enriched in the leading edge of the tumor	Cluster 4	
		Lower myogenesis and high mTOR expression signature	Clusters 5 and 7	
Cell‐surface markers	Lung cancer	Chemoresistance and poor survival	CD10^+^GPR77^+^ CAFs	130
Therapeutic profiling	Lung cancer	HGF^High^ and FGF7^High/Low^	Subtype 1	131
		HGF^Low^ and FGF7^High^	Subtype 2	
		HGF^Low^ and FGF7^Low^	Subtype 3	

*Note*: Based on the characteristic and functional studies of CAFs, CAFs are divided into different subtypes and exert diverse phenotypes and functions. Importantly, the previous studies identified four main categories of lung CAFs which are characterized by microarray technology, single‐cell RNA sequencing technique, cell‐surface markers and therapeutic profiling.

In breast cancer, CAFs subtypes can also be defined by their biomarkers, such as CD146^+^ CAFs and CD146^−^ CAFs. Compared to CD146^+^ CAFs, CD146^−^ CAFs have higher metastasis and invasion ability and lead to a poorer prognosis.^125^ In pancreatic cancer, the subtypes of CAFs can be characterized by their phenotypes, namely the myofibroblastic phenotype (myCAFs) and inflammatory phenotype (iCAFs).^126^ MyCAFs are highly expressed in αSMA and located adjacent to cancer cells, while iCAFs secrete inflammatory mediators such as interleukin‐6 (IL‐6) and are located far away from cancer cells.^126^, ^132^ Based on the heterogeneity features of CAFs, CAFs can also be divided into EMT(epithelial‐to‐mesenchymal transition)‐CAFs and PRO (proliferative)‐CAFs. These subtypes are correlated with activation of MAPK pathway and STAT3 pathway.^127^

Meanwhile, there is no standard naming for lung CAFs and the above naming in different cancer types are not translatable to lung CAFs. In lung cancer, according to histological features, Hao et al discovered two CAF subtypes from 28 NSCLC patients characterized by proliferating CAFs, namely high desmoplastic CAFs (HD‐CAF) and low desmoplastic CAFs (LD‐CAF).^128^ HD‐CAF showed a sharp rate of collagen matrix remodeling, invasion and tumor growth compared to LD‐CAFs.^128^ LD‐CAFs are associated with tumor areas with lower cellularity and less desmoplastic stromal reaction, and its predominance appears to portend a better prognosis than HDCAFs cases. Moreover, Lambrechts' group used single‐cell RNA sequencing technique to divide lung CAFs into Clusters 1, 2, 4, 5 and 7.^129^ For example, Clusters 1 and 4 were similar, but Cluster 1 showed a strong signature of EMT, an extensive repertoire of extracellular matrix proteins, and TGF‐β‐associated genes. Also, Cluster 1 was enriched within the tumor cells while Cluster 4 was enriched in the leading edge of the tumor. Cluster 2 exhibited a high level of α‐SMA and coclustered with pericytes.^129^ Clusters 5 and 7 were highly similar because of lower myogenesis and high mTOR expression signature. The differences between them were predominantly related to the expression of glycolysis genes, demonstrating metabolic differences between various CAF subsets. Su et al searched for cell‐surface markers to identify clinically‐important subtypes and found CD10^+^GPR77^+^ lung CAFs. They are related to chemoresistance and poor survival in breast and lung cancer patients.^130^ Hu et al also found three functional subtypes identified by lung cancer therapeutic profiling.^131^ Subtypes 1 and 2 CAFs have high HFG and FGF7 expression, protecting lung cancer cells by chemoresistance. HGF is a MET ligand that mediates EGFR‐inhibitor resistance via AKT and MAPK signaling.^133^, ^134^, ^135^ Subtype 3 CAFs have low HGF and FGF7 but express chemokines with chemoattractant properties for T lymphocytes and monocytes. Thus, Subtype 3 CAFs are associated with better clinical responses.

### 3.3 The main molecular markers of CAFs

**FIGURE 2:**
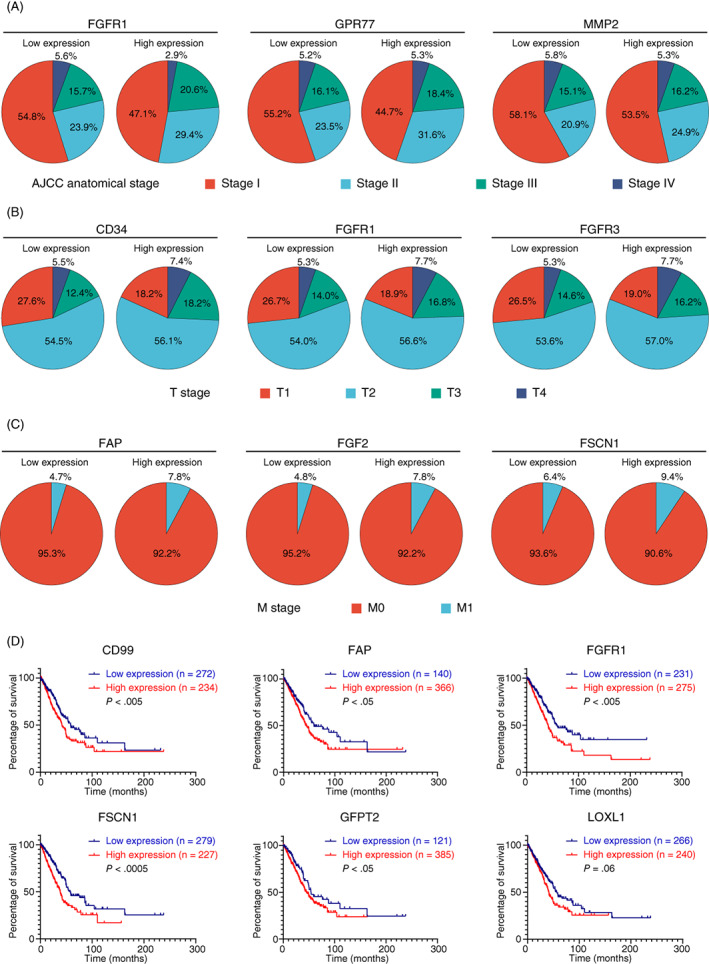
The expression and clinical significance of cancer‐associated fibroblasts (CAFs)‐related biomarkers in lung cancer. The expression level of multiple CAF biomarkers is based on (A) AJCC stages, (B) T stages and (C) M stages. The high expression of related CAF markers is associated with advanced AJCC stage, tumor invasion and distant metastasis. (D) High expression of several CAF markers is associated with unfavorable clinical outcomes in nonsmall‐cell lung cancer (NSCLC) (TCGA cohort), suggesting the promoting role of CAFs in lung cancer progression [Color figure can be viewed at wileyonlinelibrary.com]

Due to the heterogeneity of CAFs, no marker can be used as a universal and specific marker for all CAFs in different types of cancers.^136^ In addition, there are different subsets of CAFs in the tumor, increasing the difficulty in defining the appropriate markers for CAFs.^137^ In lung cancer, the most used CAF markers include, but are not limited to, alpha‐smooth muscle actin (α‐SMA) and fibroblast activation protein‐1 (FAP‐1). The reported markers are summarized in Table 2, although none of these markers are CAF‐specific, and can be expressed in other cells. Some highly expressed markers have been demonstrated to associate with advanced stages and unfavorable survival outcomes, such as FGFR1, FGF2, FAP, FSCN1 and LOXL1 (Figure 2).

**TABLE 2:** The potential biomarkers of CAFs in lung cancer

Potential biomarker	Biological functions	Promoting roles in tumors	References
αSMA	Cell contractility, structure and integrity	Tumor proliferation, immunosuppressive and impeding drug delivery	138, 139, 140
FAP‐1	ECM remodeling, fibrogenesis, serine protease activity	Tumor progression and metastasis and shaping the immunosuppressive TME	141, 142
FGFs/FGFRs	Cell proliferation, migration, differentiation and angiogenesis	Tumorigenesis	143
PDGFRβ	Receptor tyrosine kinase activity	Immunomodulation, M2 polarization and angiogenesis	142
LOXL1	Elastin, homeostasis and matrix remodeling during injury, fibrosis and cancer development	Tumorigenesis	144
VCAM1	Endothelial cell adhesion, leukocytes and mediates adhesion, signal transduction and immune responses	Growth and invasion	145
Podoplanin	Cell migration and adhesion, a specific marker of lymphatic endothelium and lymph angiogenesis	Resistance to EGFR‐TKIs, invasion, tumorigenesis and metastasis	146, 147, 148, 149, 150, 151, 152
Vimentin	Cell motility, structure and integrity	Metastasis and invasion	140, 153
GFPT2	Controls the flux of glucose into the hexosamine pathway	Metabolic reprogramming	154
MMP‐2	Degradation of ECM proteins and glycoprotein	Angiogenesis, tumor invasion and cell mobility	155, 156
CD99	Cell adhesion, migration, death, differentiation and inflammation	Migration, invasion and metastasis	157
CD34	Cell‐cell adhesion factor	Tumor vascularization	158
CD10^+^GPR77^+^	Inflammatory and enzymatic functions	Tumor formation and tumor chemosensitivity (https://www.sciencedirect.com/topics/biochemistry‐genetics‐and‐molecular‐biology/chemosensitivity)	130
CD200	Promote the protection of neurons	Promoting cancer formation and chemoresistance	159
Fascin	Regulators of the cytoskeleton	Epithelial‐to‐mesenchymal transition and cellular invasion	160

*Note*: The CAF markers in lung cancer are divided into growth factors, cytokines, ligands of immune cells, components in the extracellular matrix and other soluble factors. Each CAF marker exerts its biological and protumorigenic functions.

Abbreviations: FAP‐1, Fas‐associated phosphatase 1; FGF, fibroblast growth factors; GFPT2, GFPT2; LOXL1, lysyl oxidase‐like 1; MMP‐2, matrix metalloproteinase‐2; VCAM1, vascular cell adhesion molecule‐1; αSMA, alpha‐smooth muscle actin.

α‐SMA is widely considered as the most frequently used CAF marker.^138^, ^141^, ^161^, ^162^, ^163^, ^164^, ^165^, ^166^ CAFs which show α‐SMA expression have high collagen gel contractility (a measure of matrix remodeling capacity) and migration capacity compared to NFs.^138^, ^167^ They are associated with a high tumor Ki‐67 labeling index, lymph node metastasis, the poor 5‐year overall survival rate of the patients, and aggressive biological behavior in NSCLC.^162^, ^164^

Fibroblast‐activation protein (FAP), a cell‐surface serine protease, is a promising drug target to inhibit CAFs.^141^, ^142^, ^168^ FAP‐1 is selectively expressed by stromal mesenchymal cells and functions in wound healing, fibrotic reactions, inflammatory conditions and tumor development.^136^ Several studies reported that FAP‐1 positive CAFs exerts immunoadjuvant roles in NSCLC and FAP‐1 is considered as a molecular target in anti‐CAFs therapy. For example, a phase I dose‐escalation study with sibrotuzumab, an antibody to human FAP, in patients with advanced NSCLC, showed that sibrotuzumab explicitly binds to the tumor sites without apparent side effects.^142^, ^169^

## 4 THE PROMOTING ROLE OF CAFs ON CANCER CELLS

**FIGURE 3:**
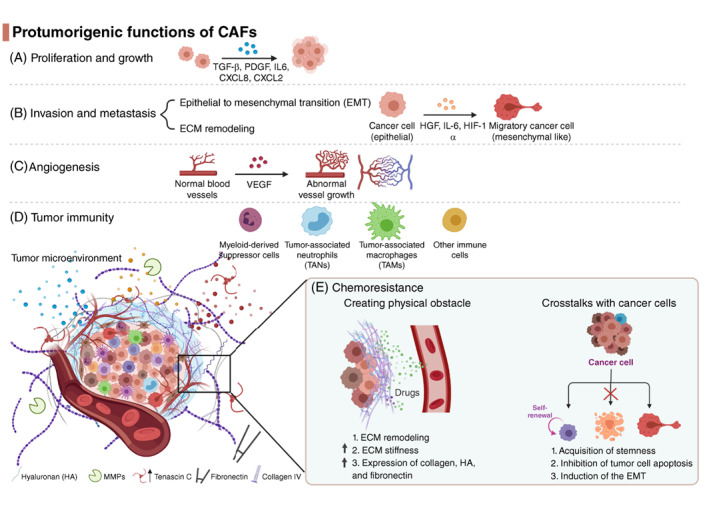
The protumorigenic roles of cancer‐associated fibroblasts (CAFs) in nonsmall‐cell lung cancer (NSCLC). CAFs secrete multiple cytokines, chemokines and growth factors to directly stimulate (A) cancer cell proliferation, (B) invasion and metastasis, (C) angiogenesis, (D) immune evasion and (E) resistance to treatment. Meanwhile, the CAFs also shape the tumor microenvironment through remodeling the extracellular matrix (ECM) to provide a chemoresistance mechanism (created with BioRender.com) [Color figure can be viewed at wileyonlinelibrary.com]

CAFs play a pivotal role in tumorigenesis and are involved in different oncogenic pathways (Figure 3). Increasing evidence has supported the protumorigenic roles of CAFs, which are summarized in Table 3.

**TABLE 3:** Multiple signaling pathways implicated in the protumorigenic functions of lung CAFs

Proteins involved in CAFs	Associated pathways in cancer cells	Coculture model/drug used	References
Proliferation, survival			
IL‐6	JAK2/STAT3	CAFs/NFs: NSCLC clinical samples Cell line: A‐549 (RRID:CVCL_0023) and SK‐MES‐1 (RRID:CVCL_0630) In vivo	168, 170
IL‐22	PI3K‐Akt‐mTOR, IL‐6‐IL‐6R	CAFs/NFs: NSCLC clinical samples Cell line: A‐549 (RRID:CVCL_0023) and NCI‐H1650 (RRID:CVCL_1483)	171
CXCL12	ERK	CAFs/NFs: NSCLC clinical samples Cell line: NSCLC‐derived neoplastic cell lines and A‐549 (RRID:CVCL_0023) In vivo	172
CLCF1	Proposed: JAK‐STAT and MAPK pathway	CAFs/NFs: Mouse fibroblast and lung adenocarcinomas clinical samples Cell line: LKR10 and LKR13 cells from Kras^LA1^ mouse, LSZ2 cells were derived through xenograft passages from Kras^LSLG12D^ mice, A‐549 (RRID:CVCL_0023) and NCI‐H1299 (RRID:CVCL_0060) In vivo	170
VCAM‐1	AKT and MAPK pathway	CAFs/NFs: Lung cancer clinical samples Cell line: A‐549 (RRID:CVCL_0023) and NCI‐H358 (RRID:CVCL_1559)	145
GGT5	N.A.	CAFs/NFs: LUAD clinical tissue Cell line: A‐549 (RRID:CVCL_0023) and ACC212102 (RRID:CVCL_D074)	173
TGF‐β	TGF‐β pathway	CAFs/NFs: Human fetal lung fibroblast 1 (HFL1) (RRID:CVCL_0298) Cell line: A‐549 (RRID:CVCL_0023)	174
HIF‐1α	NF‐kB signaling	CAFs/NFs: Mouse spontaneous LC model (TetO‐EGFRL858R; CCSP‐rtTA) and lung adenocarcinoma clinical tissues Cell line: LL/2 (LLC1) (RRID:CVCL_4358), MRC‐5 (RRID:CVCL_0440), Mouse embryonic fibroblast (MEF) cells were isolated from C57BL/6J wild‐type mice embryonic and A‐549 (RRID:CVCL_0023) In vivo	64
FGF2	FGF/FGFR pathway	CAFs/NFs: WT and Fgf9‐DT mice Cell line: TAMs and endothelial cells from Fgf9‐DT mice	143
SDF‐1	CXCR4‐mediated signaling pathway which involved NF‐κ B and Bcl‐xL	CAFs/NFs: Lung cancer clinical samples Cell line: A‐549 (RRID:CVCL_0023) and PLA‐801D (RRID:CVCL_7110)	175
Fut8	EGFR signaling	CAFs/NFs: Lung adenocarcinoma clinical sample Cell line: A‐549 (RRID:CVCL_0023), NCI‐H322 (RRID:CVCL_1556), human lung fibroblast (HLF) cells, MRC‐5 (RRID:CVCL_0440) and HFL1 (RRID:CVCL_0298) In vivo	176
miR224	Inhibiting SIRT3/AMPK and activating mTOR/HIF‐1α	CAFs/NFs: NSCLC clinical samples Cell line: A‐549 (RRID:CVCL_0023), NCI‐H1299 (RRID:CVCL_0060) and HUVEC‐C (RRID:CVCL_2959) In vivo	177
p53	N.A.	CAFs/NFs: Lung cancer clinical samples Cell line: Calu‐1 (RRID:CVCL_0608), NCI‐H460 (RRID:CVCL_0459), NCI‐H1299 (RRID:CVCL_0060) In vivo	178
FoxF1	Hedgehog signaling	Cell line: Swiss 3 T3 (NIH 3 T3) (RRID:CVCL_0594), C3H/10 T1/2 clone 8 (RRID:CVCL_0190), A‐549 (RRID:CVCL_0023), IMR‐90 (RRID:CVCL_0347) and primary murine lung fibroblasts (MLFs) were isolated from the explant out‐growth of lungs derived from wild‐type or Foxf1 heterozygous mice. In vivo Conditioned medium collected from NIH 3 T3 (RRID:CVCL_0594) and Institute for Medical Research‐90 (IMR‐90) (RRID:CVCL_0347)	179
Migration, invasion, metastasis			
Stimulation of EMT			
IL‐6	JAK2/STAT3 pathway, TGF‐β pathway	CAFs/NFs: NSCLC clinical samples, normal human lung fibroblasts (NHLF) Cell line: A‐549 (RRID:CVCL_0023), NCI‐H661 (RRID:CVCL_1577), SK‐MES‐1 (RRID:CVCL_0630) and NCI‐H358 (RRID:CVCL_1559) In vivo	168, 180
IL‐22	PI3K‐AKTmTOR pathway	CAFs/NFs: NSCLC clinical samples Cell line: A‐549 (RRID:CVCL_0023) and NCI‐H1650 (RRID:CVCL_1483)	171
Snail1 (transcription factor)	N.A.	CAFs/NFs: Lung cancer clinical samples Cell line: A‐549 (RRID:CVCL_0023), NCI‐H1299 (RRID:CVCL_0060), SPC‐A1 (HeLa derivative [endocervical adenocarcinoma], RRID:CVCL_6955) and LTEP‐a2 (HeLa derivative [endocervical adenocarcinoma], RRID:CVCL_6929)	181
HGF	HGF/IGF‐1/ANXA2 signaling	CAFs/NFs: Lung adenocarcinomas clinical samples Cell line: PC‐9 (RRID:CVCL_B260) (del E746_A750) and HCC827 (RRID:CVCL_2063) (del E746_A750)	182
IGF‐1	HGF/IGF‐1/ANXA2 signaling	CAFs/NFs: Lung adenocarcinomas clinical samples Cell line: PC‐9 (RRID:CVCL_B260) (del E746_A750) and HCC827 (RRID:CVCL_2063) (del E746_A750)	182
SRGN (a CD44‐interacting factor)	CD44/NF‐κB/claudin‐1 (CLDN1) axis	Cell line: NCI‐H1299 (RRID:CVCL_0060), NCI‐H322 (RRID:CVCL_1556), NCI‐H358 (RRID:CVCL_1559), NCI‐H23 (RRID:CVCL_1547), NCI‐H460 (RRID:CVCL_0459) and A‐549 (RRID:CVCL_0023) In vivo	183
PDGFBB	Inhibition of the PDGF‐PDGFR signaling pathway	CAFs/NFs: Lung adenocarcinomas clinical samples Cell line: A‐549 (RRID:CVCL_0023) and PC‐9 (RRID:CVCL_B260) and NCI‐H1975 (RRID:CVCL_1511)	184
PDPN	Rho‐ROCK pathway	CAFs/NFs: Lung adenocarcinomas clinical samples Cell line: A‐549 (RRID:CVCL_0023) and PC‐9 (RRID:CVCL_B260)	185
TIAM2	N.D.	CAFs/NFs: NSCLC clinical samples Cell line: A‐549 (RRID:CVCL_0023) and Medical Research Council cell strain‐5 (MRC‐5) (RRID:CVCL_0440)	186
Fascin	N.D.	CAFs/NFs: Lung adenocarcinomas clinical samples	160
HMGB1	TLR4/NF‐κB pathway	CAFs/NFs: NSCLC clinical samples Cell line: A‐549 (RRID:CVCL_0023) and NCI‐H661 (RRID:CVCL_1577)	187
Gli1 (zinc finger transcription factor)	Hedgehog signaling	CAFs/NFs: NSCLC clinical samples Cell line: NCI‐H358 (RRID:CVCL_1559)	188
SMAD3	N.A.	CAFs/NFs: NSCLC clinical samples Cell line: NCI‐H358 (RRID:CVCL_1559)	188
miR210	PTEN/PI3K/AKT pathway	CAFs/NFs: Lung adenocarcinomas clinical samples Cell line: A‐549 (RRID:CVCL_0023), NCI‐H1975 (RRID:CVCL_1511) and Bronchial Epithelium transformed with Ad12‐SV40 2B (BEAS‐2B) (RRID:CVCL_0168)	189
miR224	SIRT3/AMPK/mTOR/HIF‐1α axis	CAFs/NFs: NSCLC clinical samples Cell line: A‐549 (RRID:CVCL_0023) and NCI‐H1299 (RRID:CVCL_0060)	177
TGF‐β	TGF‐β pathway	CAFs/NFs: Normal human lung fibroblasts (NHLF) Cell line: A‐549 (RRID:CVCL_0023) and NCI‐NCI‐H358 (RRID:CVCL_1559) In vivo	180
ECM remodeling			
Vimentin	N.A.	CAFs/NFs: Lung adenocarcinomas clinical samples Transgenic mouse model	190
p53	N.A.	CAFs/NFs: Lung cancer clinical samples Cell line: Calu‐1 (RRID:CVCL_0608), NCI‐H460 (RRID:CVCL_0459) and NCI‐H1299 (RRID:CVCL_0060) In vivo	178
MMP1, 3, 10	N.A.	CAFs/NFs: Lung cancer clinical samples Cell line: NCI‐H460 (RRID:CVCL_0459)	178
Integrin α11 β 1	N.A.	CAFs/NFs: NSCLC clinical samples Cell line: NCI‐H460SM, A‐549 (RRID:CVCL_0023) and primary human lung cancer cells In vivo	191
ST8SIA2 gene	N.A.	CAFs/NFs: NSCLC clinical samples Cell line: A‐549 (RRID:CVCL_0023)	128
Angiogenesis			
CCL2/VEGFA	N.A.	CAFs/NFs: adenocarcinomas, squamous cell carcinomas and larger cell carcinomas clinical samples Cell line: A‐549 (RRID:CVCL_0023) NCI‐H460 (RRID:CVCL_0459) in vivo	192
↓miR‐1/↓miR‐206/↑miR‐31	FOXO3a/VEGF/CCL2	CAFs/NFs: adenocarcinomas, squamous cell carcinomas and larger cell carcinomas clinical samples Cell line: A‐549 (RRID:CVCL_0023) and NCI‐H460 (RRID:CVCL_0459) in vivo	192
VEGF	JAK2/STAT3 pathway	CAFs/NFs: NSCLC clinical samples Cell line: A‐549 (RRID:CVCL_0023) and NCI‐H661 (RRID:CVCL_1577) and SK‐MES‐1 (RRID:CVCL_0630)	168
bFGF	JAK2/STAT3 pathway	Cell line: A‐549 (RRID:CVCL_0023) and NCI‐H292 (RRID:CVCL_0455)	193
SDF4	ERK1/2 and p38 pathways	CAFs/NFs: HFL1 (RRID:CVCL_0298) Cell line: HUVEC‐C (RRID:CVCL_2959)	194
miR210	JAK2/STAT3 pathway	Cell line: NCI‐H1975 (RRID:CVCL_1511), A‐549 (RRID:CVCL_0023), Swiss‐3 T3 (NIH 3 T3) (RRID:CVCL_0594) and Ms‐1 (RRID:CVCL_IQ55) In vivo	75
Immunosuppression/chemoresistance			
CCL2	N.A.	CAFs/NFs: lung squamous cell carcinoma clinical samples Cell line: SW900 (RRID:CVCL_1731), NCI‐H2170 (RRID:CVCL_1535) and NCI‐H520 (RRID:CVCL_1566) and monocytes isolated from independent PBMC donors	195
↓STC‐1	N.A.	^G12D^KRAS‐ and ^V600E^BRAF‐driven mouse lung models	196
TGF‐β1	N.A.	CAFs/NFs: Lung squamous cell carcinoma clinical samples	197
IL‐6	TGF‐β‐IL‐6 Axis (Induction of the EMT and acquisition of stemness)	Cisplatin	57
SMO	Hedgehog signaling pathway (Induction of the EMT)	EGFR‐TKIs	198
SDF‐1	CXCR4‐mediated signaling pathway (Inhibition of tumor cell apoptosis)	Cisplatin	175
CCL5	Caspase‐3/BCL‐2 signaling pathway (Inhibition of tumor cell apoptosis)	Cisplatin	199
IL‐11	IL‐11R/STAT3 signaling (Inhibition of tumor cell apoptosis)	Cisplatin	199
Podoplanin	MAPK pathway and the PI3K pathway (Proposed: Induction of the EMT)	EGFR‐TKIs	200
HGF	Met/PI3K/AKT activation (Inhibition of tumor cell apoptosis)	Paclitaxel	201
IGF2	AKT/Sox2/P‐GP signals (Decrease drug retention and increase drug efflux)	Cisplatin, etoposide, vinorelbine detartrate and doxorubicin	202
IGF2	IGF‐II/IGF1R/Nanog (Acquisition of stemness)	Etoposide, docetaxel, vinorelbine detartrate and cisplatin	203
CD44	(Acquisition of stemness)	Bevacizumab and 5‐FU	204

*Note*: CAFs secrete or express proteins to promote multiple protumorigenic roles in lung cancer. The protumorigenic function is divided into four categories: proliferation and survival; migration, invasion and metastasis; angiogenesis; chemoresistance. In each category, the table highlights the signaling pathways that are involved in primary lung cancer or the coculture system of CAFs with cell lines. In the chemoresistance part, the drugs, related proteins and possible signaling pathways are also detailed summarized.

Abbreviations: CCL, C‐C chemokine ligand; CLCF1, ardiotrophin‐like cytokine factor; CXCL, CXC motif chemokine ligand; FGF, fibroblast growth factors; Fut8, fucosyltransferase 8; GGT5, gamma‐glutamyl transferase 5; HGF, hepatocyte growth factor; HIF‐1α, hypoxia‐inducible factor‐1α; HMGB1, high mobility group box 1; IGF‐1, insulin‐like growth factor; IL, interleukin; PDGF, platelet‐derived growth factor; PDPN, podoplanin; SDF‐1, stromal cell‐derived factor‐1; SMO, 7‐transmembrane protein smoothened; SRGN, sulfate proteoglycan serglycin; STC‐1, stanniocalcin‐1; TGF‐β, transforming growth factor‐beta; TIAM2, T‐cell lymphoma invasion and metastasis 2; VEGF, vascular endothelial growth factor.

### 4.1 Proliferation and growth

In two‐dimensional and three‐dimensional (3D) coculture models with lung CAFs, cancer cells grow faster than without coculture.^138^, ^161^, ^163^, ^205^ CAFs produce cytokines and growth factors that promote tumor proliferation in lung cancer cells in autocrine and paracrine manners,^206^, ^207^, ^208^, ^209^ such as C‐X‐C motif chemokine ligands including CXCL8, CXCL2,^210^ CXCL12^172^; TGF‐β,^174^ and PDGF (Figure 3A).^211^, ^212^ In addition, lung CAFs secrete Cardiotrophin‐like cytokine factor 1 (CLCF1) and IL6, which stimulate the growth of cancer cells via the JAK/STAT signaling pathway.^170^

Lung CAF‐secreted VCAM‐1 activates the AKT, JNK and P38 pathway via binding of the integrin VLA‐4 in cancer cells, thus inducing tumor growth in vivo.^145^ Various studies reported that interleukin‐22 (IL‐22) promotes the survival and tumorigenesis of cancer cells.^213^ Li et al demonstrated that CAF‐secreted IL‐22 significantly enhanced the proliferation, migration and invasion of lung cancer cells via the activation of PI3K‐Akt‐mTOR signaling.^171^ These findings suggested that the role of CAFs in activating the AKT signaling pathway is crucial for cancer cell proliferation.

Some studies have highlighted the involvement of Hh signaling involved in the activation, proliferation and invasion of CAFs and cancer cells. The biological function of Hh signaling and the associated Gli transcription factors (Gli 1‐3) promote organogenesis and lung branching morphogenesis.^214^ Olga et al reported that inhibition of Hh signaling induced a significant decrease in the proliferation of NSCLC cells by modulating cyclin D expression. In addition, NSCLC cells secreted Shh and activated Hedgehog signaling in CAFs. Lung CAFs remodel the ECM and deposit collagen, promoting cancer cells invasion and proliferation.^215^

In lung adenocarcinoma cells (LUAD), high expression of GGT5 in CAFs contributed to tumor cell proliferation and drug resistance by increasing intracellular glutathione and reducing the intracellular reactive oxygen species (ROS) level.^173^ For other cancers, increasing evidence also suggested that elevated serum gamma‐glutamyltransferase (GGT) participated in tumorigenesis and progression, such as breast cancer,^216^ gastric cancer^217^ and colorectal cancer.^218^

### 4.2 Invasion and metastasis

CAFs are a vital component in the TME and can act as a bridge between the TME and cancer cells. CAFs facilitate cancer cell crosstalk within the TME.^219^ CAFs stimulate invasion and metastasis through two main aspects, which include EMT^220^ and ECM remodeling (Figure 3B).^221^

CAFs induce EMT by secreting soluble factors.^222^, ^223^ In lung cancer, CAF‐secreted IL‐6 induces EMT programming and modulate metastasis‐related genes through the JAK2/STAT3 signaling pathway in vitro and in vivo.^168^ In our study, IL‐6 induces overexpression of EMT‐related genes and proteins, including vimentin, N‐cadherin, MMP2, MMP9 and VEGF. CAF‐secreted hepatocyte growth factor (HGF) and insulin‐like growth factor‐1 (IGF‐1) induce annexin A2 (ANXA2) expression and phosphorylation through the c‐Met pathway, resulting in EMT and EGFR tyrosine kinase inhibitors (EGFR‐TKIs) resistance in NSCLC.^182^ Also, CAF‐secreted complement 3a (C3a), a prominent tumor‐promoting factor in TME,^224^, ^225^ activates PI3K/Akt signaling. In breast cancer, The secretion of TGF‐β, HGF and PDGF by cancer cells ultimately results in EMT remodeling, invasion and metastasis.^226^

Recent studies reported that miRNAs secreted from CAFs are involved in metastasis, such as the exosomal miR‐210. It promotes EMT through targeting UPF1 to activate the PTEN/PI3K/AKT pathway in NSCLC.^189^ Besides the above signaling pathways, CAFs secrete high mobility group box 1 (HMGB1) to promote EMT through NF‐κB signaling.^187^ HMGB participates in multiple cellular processes such as invasion and angiogenesis.^227^

CAFs facilitate local invasion and metastasis of the cancer cells by biomechanically remodeling the ECM. Thus, it may be said that cancer cells invade the matrix by following the footpath of the CAFs.^228^, ^229^ CAFs synthesize structural proteins like collagen type I and IV, heparan sulfate proteoglycans, tenascin‐C, secrete connective tissue growth factors and produce digestive factors such as MMPs and plasminogen activators.^230^, ^231^ In particular, MMPs are ECM‐degrading proteases, participate in tumorigenesis and activate the inflammatory cytokines.^232^, ^233^ They are categorized into different functional subtypes and have multiple functions in the tumor stroma, including tissue invasion and intravasation, angiogenesis, regulation of inflammation and preparation of the metastatic niche.^233^ In collagen invasion assay, podoplanin (PDPN)‐expressing CAFs invade the collagen matrix, and then cancer cells invade within the footpaths created by CAFs. PDPN‐positive CAFs can be commonly found in clinical samples of lung adenocarcinoma and are also related to poor survival.^146^, ^147^, ^148^, ^149^, ^185^

### 4.3 Angiogenesis

Angiogenesis plays an important role in tumor growth and metastasis.^234^, ^235^, ^236^, ^237^ The process requires several regulatory molecules such as VEGF receptors (VEGFR),^238^ bFGF,^239^ type I collagen^240^ and fibronectin.^241^, ^242^ CAFs express these regulatory molecules to initiate angiogenesis (Figure 3C). There is also evidence that in NSCLC, when nonsmall‐cell lung cancer tumor cells are cocultured with fibroblasts, gene expressions related to tumor angiogenesis, ECM degradation, cell growth and survival are enhanced in the tumor cells.^243^

Other studies have shown that myofibroblast transformation can be induced by cisplatin and 5‐fluorouracil treatment through CCAAT/enhancer‐binding protein delta (CEBPD), thereby promoting proliferation, migration in vascular endothelial cells and angiogenesis in NSCLC cells. CEBPD elevates SDF4 (a C‐X‐C chemokine) expression in CAFs in response to cisplatin and 5‐fluorouracil treatment in HFL1 cells. SDF4 is secreted and directly interacts with CXCR4 to induce vascular endothelial growth factor D (VEGF‐D) expression for angiogenesis via ERK1/2 and p38 pathways in endothelial cells.^194^

### 4.4 Immune escaping

To achieve immune evasion, CAFs are involved in shaping the immunosuppressive TME (Figure 3D).^244^ However, the mechanism and crosstalk between the CAFs and immune cells are still to be fully elucidated. Using The Cancer Genome Atlas Lung Squamous Cell Carcinoma database, several genes are highly expressed in cases with PDPN‐expressing CAFs, including interleukin (IL)‐1A, IL‐1B, IL‐6, IL‐10, CCL2, colony‐stimulating factor 1 (CSF1), FGF2, galectin 1, PDGFA, PDGFB and TGF‐β1. Among them, TGF‐β1 is a well‐known cytokine that participated in M2 macrophage polarization and immunosuppression.^245^, ^246^ In addition, PDPN‐expressing CAFs are associated with a high number of CD204^+^ TAMs, and a low ratio of CD8^+^ T cells and FOX3^+^ T cells in immunohistochemical staining of lung adenocarcinoma specimens, suggesting that PDPN‐expressing CAFs help cancer cells escape host immunosurveillance.^197^ CAFs secrete monocyte‐ and neutrophil‐attracting chemokines and cytokines such as CCL2, CCL7, CXCL1, CXCL5, CXCL8, MIF, IL6 and VEGF in a 3D‐transwell system.^195^ MIF, IL6 and VEGF have been suggested to promote MDSC differentiation.^247^, ^248^ Significantly, CAF‐secreted CCL2 induces CCR2^+^CD14^+^ monocyte migration in chemotaxis assay and thus promotes monocyte differentiation into monocytic MDSCs.^195^ CAF‐induced MDSCs inhibit the IFNγ production of CD8^+^ T cells, thus suppressing the proliferation of CD8^+^ T cells. At the same time, they express NADPH oxidase‐2 (NOX2), which generates ROS to promote immunosuppression in lung cancer cells.^195^

To promote an immunosuppressive environment, CAFs diminish the antitumorigenic activity of natural killer cells (NK cells)^249^ To escape the attack from the immune cell, CAFs also modulate the immune checkpoints such as the programmed cell death protein 1 (PD‐1) and its ligand, programmed death‐ligand 1 (PD‐L1). PD‐1/PD‐L1 pathway suppresses the antitumor immune activity of T cells.^250^ CAF secreted cytokines such as IL‐8, osteoprotegerin (OPG) and CXCL2 can increase the expression of PD‐L1 in lung adenocarcinoma cells.^210^ More interestingly, CAFs express inhibitory ligands, including PD‐L1,^139^ PD‐L2^139^, ^251^ and FASL,^251^ which inhibit the CD8^+^ T cells in NSCLC. Apart from that, CD39^+^ T cells highly colocalized with FAP^+^ CAFs in NSCLC. These T cells and CAFs may cooperate in mediating immune escape: Activated T cells upregulate the expression of MHC, coinhibitory ligands PD‐L1 and PD‐L2, and CD73 on CAFs, increase production of IL‐6 and initiate production of IL‐27. On the other hand, CAFs enhance the level of coinhibitory receptors PD‐1, Tim3, LAG‐3 and CD39 on T cells, resulting in their transformation into tumor infiltrating T cells, and leading T‐cell apoptosis in NSCLC.^252^ The above studies suggested that CAFs play a critical role in immune checkpoint biology. However, the interplay between CAFs and cancer cells remains elusive and insufficiently delineated.

## 5 THE ROLE OF CAFs IN CHEMORESISTANCE

The two primary mechanisms used by CAFs to help cancer cells evade therapy have been demonstrated, including the physical barrier method and interplay of CAFs and lung cancer (Figure 3E).

### 5.1 The physical barrier formed by CAFs

ECM can become rigid and acts as a barrier to protect the tumor cell from chemotherapy.^253^ ECM stiffness is characterized by an aggregation of ECM proteins with hyaluronic acid (HA) at the core. CAFs enhance the expression of ECM proteins such as collagen, HA and fibronectin. Collagen and fibronectin provide resistance to tensile stress in the periphery of tumor cells.^254^, ^255^, ^256^ On the one hand, CAF‐expressed integrins α11β1 is a collagen‐binding receptor and increases ECM stiffness in NSCLC.^191^ A semisolid Matrigel‐embedded cell culture system provides a clear picture of how ECM stiffness induces chemoresistance. Lung cancer cells line A549 cells within the semisolid Matrigel matrix are arrested in the G0/G1 cell cycle, with decreased cell proliferation and invasion.^257^

### 5.2 The ligand‐receptor pathways between CAFs and cancer cells

CAF‐secreted stromal cell‐derived factor 1 (SDF‐1) enhances the chemoresistance of lung cancer cells to cisplatin by suppressing CXCR4 expression, suggesting CAFs facilitate drug resistance via the CXCR4‐mediated signaling pathway.^175^ Notably, the increased SDF‐1 was caused by a downregulation of miR‐1 which is a tumor‐suppressor microRNA and is required for transforming NFs to CAFs.^258^ On the other hand, CAFs express C‐C motif chemokine ligand 5 (CCL5) and inhibit the cisplatin‐induced apoptosis in NSCLC cells. CCL5 enhances the expression level of long noncoding RNA (lncRNA) HOX transcript antisense RNA (HOTAIR), which inhibits tumor cell apoptosis^259^, ^260^ via the caspase‐3/BCL‐2 signaling pathway.^199^

Activating EGFR mutations are common in lung cancers and can be treated by EGFR‐TKIs such as erlotinib and gefitinib. Unfortunately, most patients develop drug resistance to EGFR‐TKI. One possible explanation is that CAFs can induce EGFR‐TKI resistance. Choe et al demonstrated that coculture with CAFs induces erlotinib resistance in lung cancer cells via 7‐transmembrane protein smoothened (SMO) mediated Hh signaling. Besides, CAF‐secreted IL‐6 induces drug resistance by promoting EMT and acquiring stemness of lung cancer cells. Using a cancer tissue‐originated spheroid experiment, CAF‐secreted IL‐6 and TGF‐β contribute to tumor progression, the acquisition of stemness and drug resistance.^57^

## 6 CONCLUSION AND FUTURE PERSPECTIVES

The research about CAFs remains going on. Recently, tumor organoid studies have become popular since it is described as “cancer surrogates” that mimic the tumor's biological characteristic.^261^ An organoid derived from the patients' tumor tissue seed within the Matrigel culture system of 3D cell culture technique in vitro.^126^, ^262^, ^263^, ^264^, ^265^, ^266^, ^267^, ^268^ The Matrigel combinational culture system can simulate the ECM environment, yet it still has some limitations. The Matrigel was different from the composition of the ECM. Thus, it may not exert the entire functions and properties of fibroblasts. Moreover, the system does not contain all cell populations, such as immune cells. Thus, it is hard to investigate the crosstalk of CAFs with immune cells. Different culture systems will be developed for the deep investigation of CAFs.

Due to the heterogeneity of CAFs from the molecular aspect, several scientific and technical concerns about CAFs remain to be addressed. First, the relationship and function between CAFs at the metastatic and primary sites remains unresolved. It is unclear if primary CAFs may migrate to the metastatic site or NFs at the metastatic site are transformed to CAFs by similar cytokines produced at the primary site. Second, the classification and subtypes of CAFs are not well‐defined clearly in different cancer types. Third, it is well documented that CAFs promote tumorigenesis by remodeling the cancer cells, while how CAFs communicate with other microenvironmental components has not been clearly elucidated. Further investigations will be performed to unravel the cell‐cell chat between CAFs and endothelial, myeloid, T, B cells based on ligand‐receptor pathways. Fourth, it is urgent to develop novel research platforms for the investigation of CAFs. Apart from single‐cell RNA sequencing for the expression profiling analysis of each CAF, it also needs to develop the DNA sequencing technique in single‐cell resolution to investigate the copy number changes and mutation spectrum in CAFs.

CAFs provide a tumor‐friendly microenvironment for cancer cells and reshape their biological behaviors by cytokine secretion, ECM modification and EMT reprogramming. In this review, we summarized the molecular mechanisms and clinical significance of CAFs in NSCLC. Hopefully, the future work will shed light on developing novel therapeutic approaches by accurately targeting CAFs based on the recognized molecular mechanisms.

## AUTHOR CONTRIBUTIONS

Ka Fai To conceived the project, provided direction and guidance on the whole project. Kit Yee Wong and Alvin Ho‐Kwan Cheung drafted the manuscript. Bonan Chen and Wai Nok Chan analyzed the data and interpreted the results. Ka Fai To, Wei Kang, Kwok Wai Lo and Jun Yu reviewed the manuscript and made significant revisions. The final manuscript has been approved by all authors. The work reported in the paper has been performed by the authors, unless clearly specified in the text.

## CONFLICT OF INTEREST

The authors declare that they have no conflict of interest.
